# What is the impact of the fluid challenge technique on diagnosis of fluid responsiveness? A systematic review and meta-analysis

**DOI:** 10.1186/s13054-017-1796-9

**Published:** 2017-08-04

**Authors:** Laura Toscani, Hollmann D. Aya, Dimitra Antonakaki, Davide Bastoni, Ximena Watson, Nish Arulkumaran, Andrew Rhodes, Maurizio Cecconi

**Affiliations:** 1 General Intensive Care Unit, Adult Intensive Care Directorate, St George’s University Hospitals, NHS Foundation Trust and St George’s University of London, St James Wing, First Floor, Blackshaw Road, London, SW17 0QT UK; 2 Cristo Re Hospital, Via delle Calasanziane 25, 00167 Rome, Italy; 3 Anaesthetic Department, East Surrey Hospital, Surrey & Sussex Healthcare Trust, Canada Avenue, Redhill, Surrey, RH1 5 RH UK; 4 Cardiology Department, Broomfield Hospital, Mid-Essex Healthcare Trust, Court Road, Broomfield, Chelmsford, CM1 7ET UK; 5 Dipartimento di Medicina Sperimentale, Azienda Ospedaliero-Universitaria di Parma, Via Gramsci 14, 43126 Parma, Italy

**Keywords:** Fluid challenge, Fluid responsiveness, Fluid therapy, Fluid resuscitation

## Abstract

**Background:**

The fluid challenge is considered the gold standard for diagnosis of fluid responsiveness. The objective of this study was to describe the fluid challenge techniques reported in fluid responsiveness studies and to assess the difference in the proportion of ‘responders,’ (PR) depending on the type of fluid, volume, duration of infusion and timing of assessment.

**Methods:**

Searches of MEDLINE and Embase were performed for studies using the fluid challenge as a test of cardiac preload with a description of the technique, a reported definition of fluid responsiveness and PR. The primary outcome was the mean PR, depending on volume of fluid, type of fluids, rate of infusion and time of assessment.

**Results:**

A total of 85 studies (3601 patients) were included in the analysis. The PR were 54.4% (95% CI 46.9–62.7) where <500 ml was administered, 57.2% (95% CI 52.9–61.0) where 500 ml was administered and 60.5% (95% CI 35.9–79.2) where >500 ml was administered (*p* = 0.71). The PR was not affected by type of fluid. The PR was similar among patients administered a fluid challenge for <15 minutes (59.2%, 95% CI 54.2–64.1) and for 15–30 minutes (57.7%, 95% CI 52.4–62.4, *p* = 1). Where the infusion time was ≥30 minutes, there was a lower PR of 49.9% (95% CI 45.6–54, *p* = 0.04). Response was assessed at the end of fluid challenge, between 1 and 10 minutes, and >10 minutes after the fluid challenge. The proportions of responders were 53.9%, 57.7% and 52.3%, respectively (*p* = 0.47).

**Conclusions:**

The PR decreases with a long infusion time. A standard technique for fluid challenge is desirable.

**Electronic supplementary material:**

The online version of this article (doi:10.1186/s13054-017-1796-9) contains supplementary material, which is available to authorized users.

## Background

Intravenous fluid is one of the most commonly administered therapies for critically ill patients and is the cornerstone of haemodynamic management of patients in intensive care units (ICUs) [1]. The rationale for volume expansion is to increase the cardiac output (CO) and oxygen delivery to ultimately improve tissue oxygenation. The gold standard for assessing fluid responsiveness to guide fluid administration in critically ill patients is to perform a fluid challenge. This involves the infusion of a specific amount of intravenous fluid to assess ventricular preload reserve and subsequent systemic haemodynamic effects [2]. The volume of fluid infused must be sufficient to increase right ventricular diastolic volume and subsequently stroke volume (SV) as described by the Frank-Starling law [3]. Fluid responsiveness is conventionally defined as an increase of at least 10% to 15% in SV in response to a fluid challenge, which is a reflection of the limits of precision of the technology used [4, 5]. Patients who reach this threshold are considered ‘fluid responders’. Clinical studies have demonstrated that approximately 50% of critically ill patients who are deemed to have inadequate CO are fluid responders [6]. However, fluid responsiveness is neither a binary nor a static condition, because it depends on dynamic interaction between intravascular volume, vascular tone and ventricular function. Furthermore, fluid responsiveness may also depend on the particularities of the fluid challenge, including the type and volume of fluid as well as the administration rate.

Administration of a fluid challenge is not a standardised technique, with varying volumes, infusion rates, fluid types and durations of response. The use of different methods to estimate SV is a further confounder. Whilst different clinical conditions may require different fluid challenge techniques, there is heterogeneity in practice for the same clinical condition [6].

We hypothesise that the technique of fluid challenge affects fluid responsiveness. This may result in different clinical decisions. Either inadequate or excessive fluid administration has adverse clinical consequences, and a better understanding fluid administration is likely to improve patient management and outcome. The objective of this study was to describe the different fluid challenge techniques used in clinical trials by assessing fluid responsiveness and how the proportion of patients deemed ‘fluid-responsive’ varies according to the technique used.

## Methods

### Studies

This study was conducted following a pre-defined protocol (Additional file 1: Appendix 1). No ethical approval or patient consent was necessary for the present study. We included studies meeting the following inclusion criteria: use of a fluid challenge as a test of cardiac preload or as part of a clinical algorithm, studies performed in ICUs or operating theatres with adult patients, studies including a full description of the fluid challenge technique (volume, infusion rate, type of fluid used and timing of assessment of the haemodynamic response), studies which included a clear definition of fluid responsiveness, and studies where the numbers of responders and non-responders to the fluid challenge were stated. Only studies published as full-text articles, published in English and in an indexed journal were included. Reviews, case reports and studies published in abstract form were excluded. We excluded studies involving pregnant women and children, studies where more than one fluid challenge was performed in the same patient, studies involving passive leg raising without use of a fluid challenge technique, studies where more than one fluid type was used whilst reporting a single result, studies using a continuous infusion of fluid, and studies where the fluid responsiveness was assessed only after a period of 60 minutes or more following completion of fluid challenge. Studies reporting more than one type of fluid challenge with a full description of results for each type of fluid challenge used were included for analysis as two separate studies. Studies reporting more than one type of fluid challenge (i.e., colloids and crystalloids) without a full description of results for each type of fluid challenge were excluded from the relevant part of the analysis (i.e., type of fluid).

### Search strategy and data extraction

Three of the authors (LT, DA and DB) conducted a computerised search of the MEDLINE and Embase databases in February 2016. The terms included for the research were used in the following Boolean operators: ‘fluid challenge’ OR ‘fluid bolus’ OR ‘fluid therapy’ OR ‘fluid responsiveness’ OR ‘fluid resuscitation’ AND ‘intensive care’ OR ‘critical care’ OR ‘operative theatre’ OR ‘anaesthesia’ AND ‘stroke volume’ OR ‘cardiac output’ OR ‘cardiac index’ OR ‘stroke volume variation’ OR ‘pulse pressure variation’ OR ‘stroke pressure variation’. The search was filtered by language, the age of participants (adults) and the availability of full-text articles using the native filter function of each database used.

Titles and abstracts of the trials identified in the search were independently reviewed and pooled for further screening. The full text of each trial identified was analysed, and each reviewer compiled a list of studies that met the inclusion criteria. Each review author’s list was compared, and any disagreement was resolved through discussions until a consensus was reached among all review authors.

The following data were extracted from each study: volume of fluid used in the fluid challenge, duration of the infusion, type of fluid used, definition of *fluid responsiveness*, methodology used for the fluid responsiveness assessment, characteristics of the patients enrolled in the study, clinical environment in which the study was performed, number of patients included in the study, and percentage of ‘fluid responders’. Data were extracted independently by three authors (LT, DA and DB) and verified by another author (HDA).

**Fig. 1:**
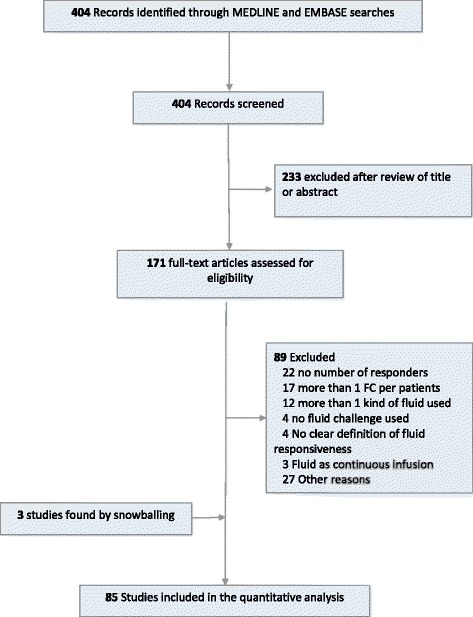
Flowchart of selection process of studies. *FC* Fluid challenge

The identification, screening and inclusion of studies in this review are summarised in a Preferred Reporting Items for Systematic Reviews and Meta-Analyses (PRISMA) diagram in Fig. 1. A PRISMA checklist is also reported in Additional file 1.

### Statistical analysis

Data were examined graphically and statistically (Shapiro-Wilk test) to understand the distribution and nature of each variable. Data are presented as mean and 95% CI when normally distributed or as median and IQR for non-parametric data. Not all the studies reported the data required for the analysis of all the outcomes. Whenever any data were missing, only those studies with the data reported for the relevant analysis were included. Not imputation technique was applied.

The primary outcome of the study was the difference in means of proportion of fluid responders (PR). The included studies were grouped into three categories on the basis of volume used for the fluid challenge: <500 ml, 500 ml and >500 ml. Studies were grouped into three categories for the duration of the fluid infusion: <15 minutes, between 15 and 30 minutes and ≥30 minutes. Cut-off values for the duration and volume of fluids infused were defined following review of the literature. The types of fluid used were grouped into two categories: colloids and crystalloids.

Two-way independent analysis of variance (ANOVA) was conducted to compare means and variances between groups using as second variable (the setting of the study: ICU vs theatre), given the potential different pathophysiology of these two groups and the potential impact on the PR. Bootstrapping was conducted using 1000 samples and bias-corrected and accelerated. When assumptions for two-way independent ANOVA were not met, one-way independent ANOVA results are reported. Post hoc test results are reported with Bonferroni correction for multiple comparisons. Statistical significance was considered at a *p* value <0.05. Statistical analysis was performed using IBM SPSS Statistics version 24 software (IBM, Armonk, NY, USA).

## Results

A total of 363 titles were identified through PubMed, and 163 were identified through Embase. After removal of duplications, 404 titles were collected for the analysis (Fig. 1). Screening by title and abstract excluded 233 studies, and 171 studies were selected for full-text assessment. Three studies were identified by snowballing. Eighty-five studies were selected for the final analysis. Two different sets of data were extracted from three studies because two different fluid challenge techniques were reported with the respective proportions of responders and non-responders. In total, 88 sets of data extracted from 85 studies with an aggregated 3601 patients were analysed (Table 1).

**Table 1:** Description of fluid challenge characteristics from the studies included in the analysis

Author	Year	Setting	N	Type of fluid	Volume	Rate or infusion time	Responders	End-point	Method of assessment	Time of assessment
Auler [20]	2008	ICU	59	Crystalloid	20 mL/Kg	20 min	39	CI > =15%	PAC	Post Hoc
Barbier [21]	2004	ICU	20	Colloids	7 mL/Kg	30 min	10	CI > =15%	TTE	Post Hoc
Belloni [22]	2007	Theatre	19	Colloids	7 mL/Kg	5 min	11	CI > 15%	PAC	Post Hoc
Biais [23]	2010	Theatre	27	Colloids	500 mL	10 min	16	CO > 15%	Vigileo	3 min
Biais [24]	2008	ICU	35	Colloids	20 mL x BMI	20 min	17	CO > =15%	Vigileo/TTE	Post Hoc
Biais [25]	2012	ICU	35	Crystalloid	500 mL	15 min	19	SV > =15%	TTE	1 min
Cannesson [26]	2009	Theatre	25	Colloids	500 mL	10 min	17	CI > 15%	Vigileo	3 min
Cannesson [27]	2008	Theatre	25	Colloids	500 mL	10 min	16	CI > =15%	PAC	4 min
Cannesson [28]	2007	Theatre	25	Colloids	500 mL	10 min	15	CI > 15%	PAC	3 min
Cecconi [29]	2012	ICU	31	Colloids	250 mL	5 min	12	SV > 15%	LiDCO	Post Hoc
Charbonneau [30]	2014	ICU	44	Colloids	7 mL/Kg	15 min	26	CI > =15%	TOE	Post Hoc
De Backer [31]	2005	ICU	60	Cryst/coll	500 & 1000 mL	30 min	33	CI > =15%	PAC	Post Hoc
De Waal [32]	2009	Theatre	18	Colloids	10 mL/Kg	10 min	15	SVI > =12%	PiCCO	Post Hoc
De Waal [32]	2009	ICU	22	Colloids	10 mL/Kg	10 min	11	SVI > =12%	PiCCO	Post Hoc
Desgranges [33]	2011	Theatre	28	Colloids	500 mL	10 min	19	CI > =15%	PAC	5 min
Dufour [34]	2011	ICU	39	Crystalloids	500 mL	5-10 min	17	SV > =15%	PiCCO	Post Hoc
Feissel [35]	2004	ICU	39	Colloids	8 mL/Kg	20 min	16	CI > 15%	TTE	1 min
Fellahi [36]	2012	ICU	25	Colloids	500 mL	15 min	14	CI > =15%	PiCCO	10 min
Fellahi [37]	2013	ICU	50	Colloids	500 mL	15 min	37	CI > =15%	PiCCO	10 min
Fellahi [38]	2012	ICU	25	Colloids	500 mL	15 min	21	CI > =15%	PiCCO	Post Hoc
Fischer [39]	2013	ICU	80	Colloids	500 mL	15 min	57	CI > =15%	PICCO	10 min
Fischer [40]	2014	ICU	50	Colloids	500 mL	15 min	41	CI > =15%	PiCCO	10 min
Fischer [41]	2013	ICU	37	Colloids	500 mL	15 min	27	CI > =15%	PiCCO	10 min
Geerts [42]	2011	ICU	24	Colloids	500 mL	N/A	17	CO > =10%	PAC	2-5 min
Guarracino [43]	2014	ICU	50	Crystalloid	7 mL/Kg	30 min	30	CI > =15%	Most Care	Post Hoc
Guerin [44]	2015	ICU	30	Crystalloid	500 mL	10 min	15	CI > =15%	PiCCO	Post Hoc
Guinot [45]	2012	Theatre	90	Crystalloid	500 mL	10 min	53	SV > 15%	TOE	Post Hoc
Guinot [46]	2015	Theatre	73	Crystalloid	500 mL	10 min	27	SV > =15%	ICG	Post Hoc
Guinot [47]	2014	Theatre	61	Crystalloid	500 mL	10 min	38	SV > 15%	ODM	1 min
Guinot [48]	2014	Theatre	42	Crystalloid	500 mL	10 min	28	SV > 15%	ODM	Post Hoc
Heenen [49]	2006	ICU	21	Cryst/coll	500 & 1000 ml	30 min	9	CO > =15%	PAC/ PiCCO	15 min
L’Hermite [50]	2013	Theatre	27	Colloid	250 mL	2-3 min	17	SVI > =10%	TOE	2 min
L’Hermite [50]	2013	Theatre	23	Crystalloid	250 mL	2-3 min	14	SVI > =10%	TOE	2 min
Hong [51]	2014	Theatre	59	Colloids	6 mL/Kg	10 min	29	CI > =15%	Vigileo	Post Hoc
Huang [52]	2008	ICU	22	Colloids	500 mL	10 ml/kg/h	10	CI > =15%	PiCCO	Post Hoc
Jung [53]	2012	A&E	26	Colloids	7 mL/Kg	30 min	17	SVI > 10%	TOE	1 min
Khwannimit [54]	2012	ICU	42	Colloids	500 mL	30 min	24	SVI > =15%	Vigileo	Post Hoc
Kuiper [55]	2013	ICU	37	Colloids	up to 200 mL	90 min	26	CI > =15%	PiCCO	Post Hoc
Kupersztych-Hagege [56]	2013	ICU	48	Crystalloid	500 mL	10 min	19	CO > =15%	PiCCO	Post Hoc
Lakhal [57]	2012	ICU	112	Colloids	500 mL	30 min	44	CO > =10%	PiCCO	1 min
Lakhal [58]	2013	ICU	130	Crystalloid	500 mL	30 min	48	CO > 10% or CO >15%	PiCCO	1 min
Lamia [59]	2007	ICU	24	Crystalloid	500 mL	15 min	13	SVI > =15%	TTE	Post Hoc
Lanspa [60]	2012	ICU	14	Crystalloid	10 mL/Kg	<20 min	5	CI > =15%	TTE	Post Hoc
Lee [61]	2007	Theatre	20	Colloids	7 mL/Kg	1 mL/Kg/min	11	SVI > 10%	TOE	1 min
Loupec [62]	2011	ICU	40	Colloids	500 mL	10 min	21	CO > =15%	TTE	Post Hoc
Machare-Delgado [63]	2011	ICU	25	Crystalloid	500 mL	10 min	8	SV > =10%	TTE	30 min
Mahjoub [64]	2009	ICU	35	Colloids	500 mL	30 min	23	SV > =15%	TTE	5 min
Maizel [65]	2007	ICU	34	Crystalloid	500 mL	15 min	17	CO > =10%	TTE	Post Hoc
Mallat [66]	2015	ICU	49	Colloids	100 + 500 mL	15 min	22	CI > =15%	PiCCO	Post Hoc
Mekontso-Dessap[67]	2006	ICU	37	Colloids	500 mL	15-30 min	15	CI > =15%	PAC	Post Hoc
Monge [68]	2009	ICU	30	Colloids	500 mL	30 min	11	SVI > =15%	Vigileo	1 min
Monge [69]	2009	ICU	38	Colloids	500 mL	30 min	19	SVI > =15%	Vigileo	1 min
Monnet [70]	2011	ICU	228	Crystalloid	500 mL	20 min	142	CO > =15%	PiCCO	Post Hoc
Monnet [71]	2012	ICU	38	Crystalloid	500 mL	30 min	16	SVI > =15%	Nexfin	1 min
Monnet [72]	2013	ICU	51	Crystalloid	500 mL	30 min	25	CI > =15%	PiCCO	Post Hoc
Monnet [73]	2006	ICU	71	Crystalloid	500 mL	10 min	37	CO > =15%	TOE	Post Hoc
Monnet [74]	2012	ICU	39	Crystalloid	500 mL	30 min	17	CI > =15%	PiCCO	Post Hoc
Monnet [75]	2012	ICU	54	Crystalloid	500 mL	20 min	30	CI > =15%	PiCCO	Post Hoc
Moretti [76]	2010	ICU	29	Colloids	7 mL/kg	30 min	17	CI > =15%	PiCCO	Post Hoc
Muller [77]	2010	ICU	57	Cryst /coll	250 or 500 mL	999 mL/h	41	SVI > =15%	PAC/ PiCCO	10 min
Natalini [78]	2006	ICU	22	Colloids	500 mL	30 min	13	CI > =15%	PAC	Post Hoc
Oliveira-costa [79]	2012	ICU	37	Cryst&coll	500 & 1000 mL	30 min	17	CI > =15%	PAC	Post Hoc
Perner [80]	2006	ICU	30	Crystalloid	500 mL	30 min	14	CI > 10%	PiCCO	Post Hoc
Pierrakos [81]	2012	ICU	29	Crystalloid	1000 mL	30 min	13	CI > 10%	PAC	Post Hoc
Pierrakos [81]	2012	ICU	22	Colloids	500 mL	30 min	11	CI > 10%	PAC	Post Hoc
Pranskunas [82]	2013	ICU	50	Cryst/coll	500 mL	30 min	34	SVI > =10%	PiCCO /PAC	Post Hoc
Preau [83]	2010	ICU	34	Colloids	500 mL	30 min	14	SVI > =15%	TTE	Post Hoc
Royer [84]	2015	ICU	16	Crystalloid	500 mL	30 min	9	CO > =15%	TTE	Post Hoc
Saugel [85]	2013	ICU	24	Crystalloid	7 mL/Kg	30 min	7	CI > =15%	PICCO	Post Hoc
Siswojo [86]	2014	Theatre	29	Colloids	500 mL	5 min	17	SVI > =10%	TOE	1 min
Smorenberg [87]	2013	ICU	32	Colloids	250 mL	1000 ml/h	14	SVI > 10%	PAC	30 min
Soltner [88]	2010	ICU	40	Colloids	500 mL	20 min	16	CI > 12%	PAC	Post Hoc
Song [89]	2014	Theatre	40	Colloids	6 mL/Kg	N/A	23	SVI > =15%	PAC	1 min
Sturgess [90]	2010	ICU	10	Colloids	250 mL	15 min	4	SV > 15%	USCOM	5 min
Suehiro [91]	2012	ICU	80	Crystalloid	500 mL	30 min	38	CI > =15%	PAC	Post Hoc
Taton [92]	2013	ICU	33	Cryst/coll	500-1000 mL	15-30 min	17	CO > =10%	TTE / Nexfin	1 min
Vallee [93]	2005	ICU	51	Colloids	4 mL/Kg	15 min	20	CO > 15%	TOE	Post Hoc
Vallee [94]	2009	ICU	84	Colloids	6 mL/Kg	30 min	39	CI > 15%	PiCCO	Post Hoc
van Haren [95]	2012	ICU	12	Cryst/coll	250 mL	15 min	4	CI > 10%	PiCCO	30 min
Yazigi [96]	2012	Theatre	60	Colloids	7 mL/Kg	20 min	41	SVI > =15%	PAC	2 min
Viellard-Baron [97]	2004	ICU	66	Colloids	10 mL/Kg	30 min	20	CI > =11%	TTE	Post Hoc
Vistisen [98]	2009	ICU	23	Colloids	500 mL	90 min	17	CI > 15%	PAC	Post Hoc
Wiesenack [99]	2005	Theatre	20	Colloids	7 mL/Kg	1 mL/kg/min	13	SVI > =20%	PiCCO	1 min
Wiesenack [100]	2005	Theatre	21	Colloids	7 mL/Kg	1 mL/Kg/min	19	SVI > =10%	PAC	12 min
Wilkman [101]	2014	ICU	20	Colloids	6 mL/Kg	N/A	6	CO > 15%	TOE	1 min
Xiao-Ting [102]	2015	ICU	48	Crystalloid	500 mL	15 min	34	CI > =10%	PiCCO	Post Hoc
Zimmermann [103]	2010	Theatre	20	Colloids	7 mL/Kg	1 mL/Kg/min	15	SVI > =15%	Vigileo	1 min

*ICU* intensive care unit, *CO* cardiac output, *CI* cardiac index, *SV* stroke volume, *SVI* stroke volume index, *TOE* trans-oesophageal echocardiography, *TTE* trans-thoracic echocardiography, *PAC* pulmonary artery catheter, *min* minutes, *USCOM* transcutaneous aortic Doppler, *ICG* impedance cardiography, *ODM* oesophageal Doppler monitoring, *N/A* data not available, *Post Hoc* immediate reading

The definition of positive response to a fluid challenge varies substantially across studies (Additional file 1: Figure S1). Physiological parameters used to assess fluid response include cardiac index (47.5%), CO (17.1%), SV (11.0%) and stroke volume index (24.3%). The increment from baseline measurements in physiological parameters deemed to have a positive response to a fluid challenge was either 10% (25.5% of studies) or 15% (74.5% of studies). The most frequent definition of a positive response to a fluid challenge was an increase in cardiac index of at least 15% from baseline (*n* = 33 [40.2%]). CO was estimated using several different technologies (Additional file 1: Figure S2), with pulse index continuous CO (PiCCO; PULSION Medical Systems, Feldkirchen, Germany) used most frequently (31.7% of studies), followed by the pulmonary artery catheter (PAC; 22% of studies) (Table 1). There was a higher percentage of responders in studies performed in the operating room (63.4%, 95% CI 58.3–68.4) than in the ICU (51.5%, 95% CI 48.2–54.8, *p* < 0.001).

### Volume of fluid challenge

The volumes of fluid administered for the fluid challenge varied from <500 ml (*n* = 8 [12.7%]) to 500 ml (*n* = 50 [79.4%]) and >500 ml (*n* = 5 [7.9%]). Twenty-four studies were excluded from this analysis because the volume was described as milligrams per kilogram and the participants’ body weight was not reported. The estimated mean PR values were 54.4% (95% CI 46.9–62.7) among patients receiving <500 ml, 57.2% (95% CI 52.9–61.0) among patients receiving 500 ml and 60.5% (95% CI 35.9–79.2) among patients receiving >500 ml. There was no difference in the PR values between groups of patients receiving different volumes of fluid challenges [*F*(2,57) = 0.35, *p* =0.71] (Additional file 1: Figure S3). The PR observed in studies where the fluid was prescribed as a fixed volume (*n* = 63 [72.4%]) and where fluid volume was adjusted for body weight (*n* = 24 [27.6%]) was similar [*F* (1,83) = 0.02, *p* = 0.88].

### Type of fluid

Twenty-six (35%) studies used crystalloids, and 50 (65%) used colloids. Nine studies were excluded from the analysis because they used both types of fluids. Among patients receiving crystalloids, 53.5% (95% CI 45.4–58.5) were responders, as compared with 59.0% (95% CI 55.5–62.9) in the group receiving colloids (Additional file 1: Figure S4). The type of fluid used did not affect the proportion of patients responding to a fluid challenge [*F*(1,76) = 2.19, *p* = 0.14].

### Duration of infusion

**Fig. 2:**
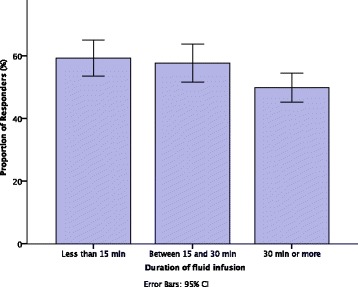
Comparison of the proportion of responders (%) by duration of the infusion used for the fluid challenge. Planned contrast analysis revealed a significant difference between the third group (≥30 minutes) and the other two groups

The time of infusion was <15 minutes in 24 studies (27.3%), between 15 and 29 minutes in 26 studies (29.5%), and ≥30 minutes in 29 studies (33%). Nine studies (10.2%) did not report duration of infusion. Where the fluid challenge was administered for <15 minutes, between 15 and 29 minutes, and >30 minutes, the proportions of patients deemed to be fluid responders were 59.2% (95% CI 54.2–64.1), 57.7% (95% CI 52.4–62.4), and 49.9% (95% CI 45.6–54) respectively. The duration of the fluid infusion affects the proportion of fluid responders [*F*(2,73) = 3.63, *p* = 0.03] (Fig. 2). The PR to a fluid challenge given in ≥30 minutes was lower than the PR when the fluid challenge was given in <15 minutes (*p* = 0.045). The proportion of patients responding to a fluid challenge that was administered in <15 minutes and between 15 and 30 minutes was similar (*p* = 1.0).

### Timing of assessment

**Fig. 3:**
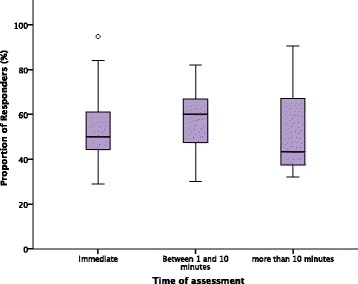
Comparison of the proportion of responders (%) by assessment time after the fluid challenge

The assessment of response to a fluid challenge was at the point of administration (*n* = 50 [58.1%]), between 1 and 10 minutes (*n* = 31 [36.8%]), or >10 minutes (*n* = 5 [5.8%]) after completion of the fluid challenge. Where fluid responsiveness was assessed at the point of administration, between 1 and 10 minutes, and >10 minutes after completion of the fluid challenge, 53.9% (95% CI 49.8–57.7), 57.7% (95% CI 52.9–62.7), and 52.3% (95% CI 32–90.5) of patients had a positive response, respectively. The time of assessment of fluid response did not affect the PR [*F*(2,80) = 0.76, *p* = 0.47] (Fig. 3).

## Discussion

We demonstrate that the duration of the fluid infusion in a fluid challenge has a significant influence on fluid responsiveness. This confirms our hypothesis that the proportion of patients deemed to respond to a fluid challenge is influenced by the characteristics of a fluid challenge technique, in addition to intravascular filling, vascular tone or ventricular contractility. Other aspects of the fluid challenge, including the volume, type of fluid or assessment time, do not affect the proportion of patients who are fluid responders. Currently, no consensus exists on how to perform an effective fluid challenge. This study highlights the need for a standardised technique for research and clinical purposes.

Fluid challenge is one of the commonest interventions in critical care medicine. A recent international observational study [6] including 2279 patients from 311 centres highlighted the variability in this intervention. In contrast to our results, crystalloids were more frequently used (74.0%), with balanced solutions used in most of cases (53.3%). The study was undertaken following the publication of large, randomised clinical trials advocating the use of crystalloids over colloids [7–10]. Up to two to three times as much crystalloid as colloid may be required to maintain intravascular volume, owing to differences in intravascular half-life [11]. Fluid challenges consisting of colloids compared with crystalloids are associated with a more linear increase in cardiac filling and SV compared with crystalloids [12].

However, the theoretical benefits of colloids over crystalloids in critically ill patients with altered endothelial permeability have not been borne out in clinical trials. Starch-based solutions are associated with increased rates of acute kidney injury and coagulopathy compared with crystalloid solutions [7, 8, 13]. Human albumin solution is associated with a poorer prognosis in patients with traumatic brain injury [14] and is not associated with any survival benefit compared with colloids in patients with sepsis [15]. We did not find any difference in PR by the type of fluid used for a fluid challenge. If the time of assessment of fluid responsiveness is immediately after fluid infusion or in the first minutes, it is unlikely that the type of fluid would make any difference, because in both cases (colloids/crystalloids) it is likely that a big proportion of the volume infused will remain in the intravascular compartment. If the assessment of fluid responsiveness were performed later, it would be possible to observe some differences because theoretically colloids remain longer in the intravascular space than crystalloids do. This would require further investigation.

Consistent with a recent large observational study [6], the most common volume of fluid used for a fluid challenge was 500 ml. However, there was significant variability in the volume of fluid used. The total volume of fluid administered to determine fluid responsiveness varies widely, from 4 to 20 ml/kg or 100 to 1000 ml. Whilst fluid challenge with larger volumes may have serious clinical consequences, such as pulmonary oedema, very small volumes may not represent a cardiovascular challenge. The clinical challenge lies in determining the optimal volume of fluid required to optimise cardiac performance and tissue perfusion. The effect of the volume of fluid challenge was recently investigated by our group [16]. Eighty patients were administered four different volumes as fluid challenges (1, 2, 3 and 4 ml/kg of crystalloids) over 5 minutes. Pmsf-arm, a surrogate of the mean systemic filling pressure (Pmsf), was measured. Pmsf itself is a measure of effective intravascular filling independent of cardiac function [17]. This technique has been shown to be precise for a change of 14% from baseline [18]. The minimal volume required to achieve an increment of 14% was 4 ml/kg. Importantly, the dose of fluids used affects the change in CO and consequently the proportion of patients considered to be responsive to a fluid challenge. Differences in the volume of fluid required to achieve a positive fluid response between this study and other studies in this meta-analysis may be explained by the heterogeneity in the methods used for estimating CO, thresholds defining a positive response, patient case mix and illness severity.

The optimal rate of fluid infusion is unknown. The researchers in the Fluid Challenges in Intensive Care (FENICE) study [6] reported a median infusion time of 24 minutes to administer a fluid challenge. Our results suggest that the duration of the fluid infusion has a significant effect on observed fluid responders. An infusion time <30 minutes is more effective in detecting fluid responders than infusion times >30 minutes. These results are consistent with our understanding of cardiovascular physiology, where a rapid intravenous fluid bolus will rapidly increase venous return to increase right ventricular end-diastolic volume. A slower rate of infusion, however, would result in a lower increase of venous return and result in a lower rise in SV, thus becoming less effective. Prospective clinical studies are warranted before these findings can be incorporated into routine clinical practice.

Pooled data in this meta-analysis indicate that the timing of assessment of a fluid challenge does not have a significant impact on detecting a positive response. This is in contrast to previous work by our group in which the haemodynamic effect of a 250-ml crystalloid fluid challenge was almost completely dissipated after 10 minutes from the end of the fluid challenge [19]. In this meta-analysis, many studies used PAC as a method to estimate CO, which cannot accurately detect immediate changes in SV. This makes it more challenging to study the immediate physiological effect of the fluid challenge on SV. A more sustained response would intuitively be clinically favourable. However, this is likely to be influenced by the patient’s underlying pathophysiology in addition to the fluid challenge technique itself. In this study, it is possible to comment only on the physiological effect of the fluid challenge, because the clinical effect is beyond the scope of this review. Another possible explanation for the discrepancy in results is the distribution of studies between categories of the assessment time: only five studies reported a time of assessment after 10 minutes, which is the time point at which we have previously observed complete dissipation of the haemodynamic effect of the fluid challenge.

As with all retrospective observational studies, the data presented must be interpreted in the context of its limitations. There is likely to be significant heterogeneity in the patient case mix, illness severity and overall management. Different permutations of the rate of fluid administered, the type and volume of fluid, method of haemodynamic assessment, threshold for definition of responsiveness, and the time of assessment of fluid challenge does not allow any strong conclusions to be made. Furthermore, we have not accounted for the different methods of haemodynamic monitoring used. However, we highlight the heterogeneity in practice of this commonly applied technique and the need for further investigation to elucidate the clinical effect of the different aspects of a fluid challenge.

## Conclusions

The proportion of patients who respond to a fluid challenge is dependent on the particularities of the technique used. A rapid infusion of fluid volume increases the proportion of patients with a positive response. However, the type and volume of fluid or the time of assessment does not appear to have any effect on the detection of fluid responders. This study highlights that standardisation of the fluid challenge technique is needed for contextualisation of clinical trial data and patient management.

## Additional file

Additional file 1: Study protocol, PRISMA checklist and additional figures. (PDF 674 kb)

## Abbreviations

ANOVA: Analysis of variance
BMI: Body mass index
CO: Cardiac output
FC: Fluid challenge
ICG: Impedance cardiography
ICU: Intensive care unit
N/A: Not available
ODM: Oesophageal Doppler monitoring
PAC: Pulmonary artery catheter
Pmsf: Mean systemic filling pressure
PR: Proportion of responders
PRISMA: Preferred Reporting Items for Systematic Reviews and Meta-Analyses
SV: Stroke volume
SVI: Stroke volume index
TOE: Trans-oesophageal echocardiography
TTE: Trans-thoracic echocardiography
USCOM: Transcutaneous aortic Doppler ultrasonic cardiac output monitor
