# Supplementary material for: What is the impact of the fluid challenge technique on diagnosis of fluid responsiveness? A systematic review and meta-analysis
Source: Crit Care. 2017 Aug 4;21:207. doi: 10.1186/s13054-017-1796-9 (PMC5543539; doi:10.1186/s13054-017-1796-9)

The fluid challenge technique and diagnosis of fluid responsiveness: a systematic review and meta-analysis

Laura Toscani (MD), Hollmann D. Aya (PhD), Dimitra Antonakaki (MD), Davide Bastoni (MD), Ximena Watson (MD), Nish Arulkumaran (PhD), Andrew Rhodes (MD(Res)), Maurizio Cecconi (MD(Res)).

Additional File

Table of Contents

eAppendix 1 Study Protocol 3

PRISMA 2009 Checklist 11

eFigures 14

eAppendix 1 Study Protocol

Study Title: A systematic review and metanalysis of fluid challenge in anaesthesia and intensive care.

JREO Reference Number: N/A

Ethics Ref: N/A

Date and Version No: v 1. 26th October 2015

Chief Investigator: Maurizio Cecconi

Investigators: Laura Toscani, Dimitra Antonakaki, Davide Bastoni, Hollmann D. Aya, Andrew Rhodes

Sponsor: St George's University of London/St George's Healthcare NHS Trust

Funder: SGUL internal grant

The investigators declare no potential conflicts of interest.

Confidentiality Statement

This document contains confidential information that must not be disclosed to anyone other than the Sponsor, the Investigator Team, host organisation, and members of the Research Ethics Committee, unless authorised to do so.

Background

Fluids are one of the most commonly used therapies in the hemodynamically unstable patients and represent the cornerstone of hemodynamic management in intensive care units (ICUs) [1].

The aim of volume expansion is to increase cardiac index and oxygen delivery and to improve tissue oxygenation. However, this occurs only in a situation of preload dependency. The gold standard to evaluate fluid responsiveness and guide fluid administration in critically ill or surgical patients is to perform a fluid challenge. This is a dynamic test of the cardiovascular system that assesses the preload reserve of the patient[2].

The volume of fluid given for this test must be sufficient to stretch the right ventricle, increasingthe diastolic volume. Under this condition, SV may increase according to Frank–Starling's law [3].

The response is considered positive when SV or CO increases. Fluid responsiveness has been arbitrarily defined as an increase of at least 10 -15% in cardiac output in response to a 500 mlbolus fluid challenge [4].

Patients who reach this target are defined "fluid responders".

In "fluid responders" patients further fluids can be given in a controlled manner, repeating the fluid challenge so long as there is a positive response (SV maximization). This approach avoids fluid overload [2].

However, responsiveness is not a categorical condition but a temporal situation that may also depend on the volume of fluid given in the fluid challenge and on the administration rate.

Recently the FENICE study has highlighted the huge variability in the current practice regarding the fluid challenge. Different techniques are used around the globe in terms of volume, infusion rate and type of fluid.

This can reflect the presence of controversies in current guidelines. However, while it is true that different conditions may require different techniques, data show extensive variability even within the same clinical condition [5]. These variables could be very important in determinate the response to a fluid challenge.

Clinical studies have consistently demonstrated that about 50% of hemodynamically unstable critically ill patients are volume responsive [6].

Hypothesis & research question

The research question is: How is a fluid challenge performed in the literature and how the different aspects of the fluid challenge technique impact the discrimination between responders and not responders?

We hypothesised that the percentage of responders and not responders changes depending on the technique used for a fluid challenge.

Aims

Aim of this systematic review and metanalysis is to describe the fluid challenge technique in fluid responsiveness studies and clinical trials and to assess if there is a difference in number or percentage of "fluid responders" and "fluid not responders" depending on the fluid challenge technique used in terms of type of fluid, volume and infusion rate.

We will also assess how/if the definition of fluid responsiveness and the device used to measure the chosen parameter affect the number of responders and not responders.

Methods

Eligibility Criteria for studies studies

Inclusion criteria

Studies fulfilling all the following inclusion criteria will be included in the metaanalysis:

- Studies using a fluid challenge as a test of cardiac preload or as part of a clinical algorithm.
- Studies performed in intensive care unit or operative theatre.
- The studies must include a description of the fluid challenge technique reporting the volume, infusion rate, type of fluid used, and timing of assessment of the haemodynamic response.
- The definition of fluid responsiveness used for the study must be reported as well as the percentage or number of responders and non-responders.

Exclusion criteria

Studies will be excluded if any of the following criteria are met:

- Studies on a population of pregnant women or children.
- More than one fluid challenge performed in the same patient.
- Studies using passive leg raising as the main test of preload instead of the fluid challenge
- Studies using more than one fluid type whilst reporting a single result for the whole study.
- Studies using a continuous infusion of fluid or where the fluid responsiveness was only assessed after a period of 60 minutes or more following completion of fluid infusion.

Participants

We will include studies performed in a population of adults patients admitted in Intensive Care Unit or operative theatre, who received a fluid challenge and the consecutive assessment of thefluid responsiveness.

Comparison

We will divide the studies whose match the inclusion criteria in groups (categories) for type of fluid, volume used, duration of infusion and assessment time.

We will compare the number of “fluid responders” and “fluid non responders” patients for each group of studies. This will allow us to have a percentage of “fluid responders” and “fluid non responders” patients for each groups.

Primary outcome

The primary outcome of the study is the mean proportion fluid responders after a fluid challenge with less than 500 mL compared with 500 mL or more.

Secondary outcome is the mean proportion of fluid responders after a fluid challenge according to different types of volume, infusion rates and assessment times.

Search strategy

Electronic searching

We will search the Medline and EMBASE databases. We will include in our research the following terms:

- “fluid challenge” OR “fluid bolus” OR “fluid therapy” OR “fluid responsiveness” OR “fluid resuscitation”

AND

- “intensive care” OR “critical care” OR “operative theatre” OR “anaesthesia”

AND

- “stroke volume” OR “cardiac output” OR “cardiac index” OR “stroke volume variation” OR “pulse pressure variation OR “stroke pressure variation”.

Our search will be limited by language (English), age of participants (adults) and availability of full text article (if only the abstract of the article has been published it will be not included in our review).

We will use the database’s filters in order to follow our research strategy.

Reference lists

The reference lists of articles will be searched for relevant articles. The ones meeting all the inclusion criteria and no exclusion criteria will be included in the study.

Data collection and analysis

A summary of the identification, screening and inclusion of studies in this review will be presented as a PRISMA (Preferred Reporting Items for Systematic Reviews and Meta-analyses) flowchart.

Selection of studies

Two review authors (TL and AD) will independently screen and select studies for possible inclusion in the study. First, the titles and abstracts of trials identified from the search will be independently reviewed and pooled for further screening. Secondly, each review author will independently examine the full text of all trials that were identified from the title and abstract screens. Each reviewer will compile a list of studies that meet the inclusion criteria.

The contents of each review author's list will be compared, and any disagreement will be resolved by discussion and consensus between all of the review authors.

Data extraction

From each study will be extracted:

-The volume of fluid used in the fluid challenge

-The infusion rate of the fluid

-The type of fluid used

-The definition of "fluid responsiveness" used

-The methodology used for the fluid responsiveness assessment

-The characteristics of the patient enrolled in the study

-The clinical environment in which the study has been performed

-The number of patients included in the study

-The percentage of “fluid responders” and “fluid non responders”.

References

1. Myburgh JA, Mythen MG. Resuscitation fluids. N Engl J Med. 2013;369:2462–2463. doi: 10.1056/NEJMra1208627

2. Cecconi M, Parsons AK, Rhodes A. What is a fluid challenge? Curr Opin Crit Care 2011; 17:290–295

3. Patterson SW, Starling EH. On the mechanical factors which determine the output of the ventricles. J Physiol 1914; 48:357–379.

4. Michard F, Boussat S, Chemla D, et al. Relation between respiratory changes in arterial pulse pressure and fluid responsiveness in septic patients with acute circulatory failure.Am J Respir Crit Care Med 2000; 162:134–138

5. Cecconi, Maurizio, et al. "Fluid challenges in intensive care: the FENICE study." Intensive care medicine 41.9 (2015): 1529-1537.

6. Marik PE, Cavallazzi R, Vasu T, et al. Dynamic changes in arterial waveform derived variables and fluid responsiveness in mechanically ventilated patients. A systematic review of the literature. CritCare Med 2009; 37: 2642–7.

PRISMA 2009 Checklist

Section/topic	#	Checklist item	Reported on page #
**TITLE**			
Title	1	Identify the report as a systematic review, meta-analysis, or both.	1
**ABSTRACT**			
Structured summary	2	Provide a structured summary including, as applicable: background; objectives; data sources; study eligibility criteria, participants, and interventions; study appraisal and synthesis methods; results; limitations; conclusions and implications of key findings; systematic review registration number.	3
**INTRODUCTION**			
Rationale	3	Describe the rationale for the review in the context of what is already known.	5
Objectives	4	Provide an explicit statement of questions being addressed with reference to participants, interventions, comparisons, outcomes, and study design (PICOS).	6
**METHODS**			
Protocol and registration	5	Indicate if a review protocol exists, if and where it can be accessed (e.g., Web address), and, if available, provide registration information including registration number.	6
Eligibility criteria	6	Specify study characteristics (e.g., PICOS, length of follow-up) and report characteristics (e.g., years considered, language, publication status) used as criteria for eligibility, giving rationale.	6
Information sources	7	Describe all information sources (e.g., databases with dates of coverage, contact with study authors to identify additional studies) in the search and date last searched.	6
Search	8	Present full electronic search strategy for at least one database, including any limits used, such that it could be repeated.	5 in supplement
Study selection	9	State the process for selecting studies (i.e., screening, eligibility, included in systematic review, and, if applicable, included in the meta-analysis).	7
Data collection process	10	Describe method of data extraction from reports (e.g., piloted forms, independently, in duplicate) and any processes for obtaining and confirming data from investigators.	7

Data items	11	List and define all variables for which data were sought (e.g., PICOS, funding sources) and any assumptions and simplifications made.	7 (paper) 9 (supplement)
Risk of bias in individual studies	12	Describe methods used for assessing risk of bias of individual studies (including specification of whether this was done at the study or outcome level), and how this information is to be used in any data synthesis.	
Summary measures	13	State the principal summary measures (e.g., risk ratio, difference in means).	8
Synthesis of results	14	Describe the methods of handling data and combining results of studies, if done, including measures of consistency (e.g., I^2) for each meta-analysis.	8

Section/topic	#	Checklist item	Reported on page #
Risk of bias across studies	15	Specify any assessment of risk of bias that may affect the cumulative evidence (e.g., publication bias, selective reporting within studies).	N/A
Additional analyses	16	Describe methods of additional analyses (e.g., sensitivity or subgroup analyses, meta-regression), if done, indicating which were pre-specified.	N/A
**RESULTS**			
Study selection	17	Give numbers of studies screened, assessed for eligibility, and included in the review, with reasons for exclusions at each stage, ideally with a flow diagram.	Figure 1
Study characteristics	18	For each study, present characteristics for which data were extracted (e.g., study size, PICOS, follow-up period) and provide the citations.	Table 1 & references
Risk of bias within studies	19	Present data on risk of bias of each study and, if available, any outcome level assessment (see item 12).	N/A
Results of individual studies	20	For all outcomes considered (benefits or harms), present, for each study: (a) simple summary data for each intervention group (b) effect estimates and confidence intervals, ideally with a forest plot.	N/A
Synthesis of results	21	Present results of each meta-analysis done, including confidence intervals and measures of consistency.	9 - 10
Risk of bias across studies	22	Present results of any assessment of risk of bias across studies (see Item 15).	N/A
Additional analysis	23	Give results of additional analyses, if done (e.g., sensitivity or subgroup analyses, meta-regression [see Item 16]).	N/A
**DISCUSSION**			

Summary of evidence	24	Summarize the main findings including the strength of evidence for each main outcome; consider their relevance to key groups (e.g., healthcare providers, users, and policy makers).	11
Limitations	25	Discuss limitations at study and outcome level (e.g., risk of bias), and at review-level (e.g., incomplete retrieval of identified research, reporting bias).	13
Conclusions	26	Provide a general interpretation of the results in the context of other evidence, and implications for future research.	14
**FUNDING**			
Funding	27	Describe sources of funding for the systematic review and other support (e.g., supply of data); role of funders for the systematic review.	N/A

From: Moher D, Liberati A, Tetzlaff J, Altman DG, The PRISMA Group (2009). Preferred Reporting Items for Systematic Reviews and Meta-Analyses: The PRISMA Statement. PLoS Med 6(6): e1000097. doi:10.1371/journal.pmed1000097

For more information, visit: **www.prisma-statement.org**.

eFigures

eFigure 1 Distribution of definitions of “fluid responders” across studies. CI cardiac index, CO cardiac output, SV stroke volume, SVI stroke volume index.

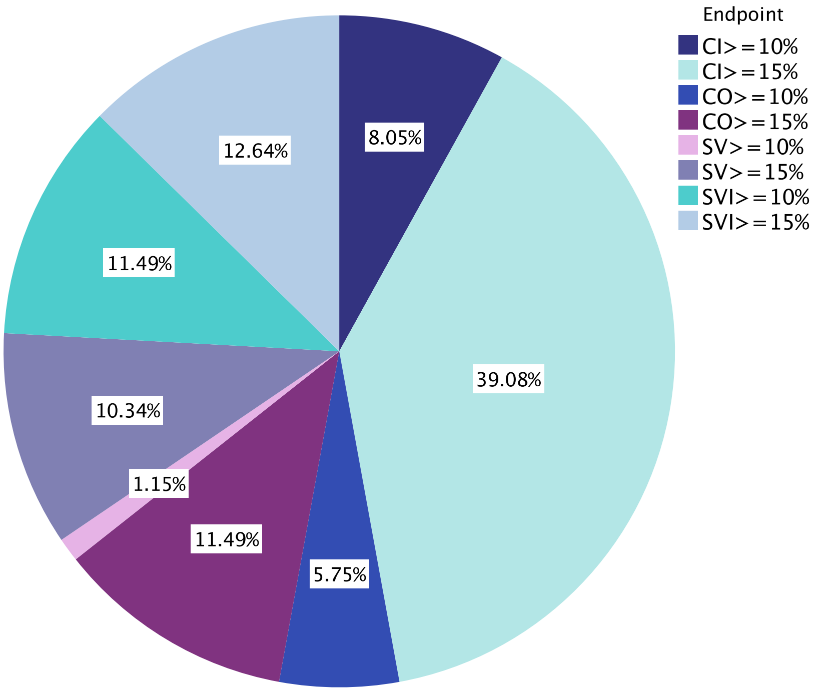

eFigure 2 Distribution of methods used to measure cardiac output across studies. PiCCO pulse index continuous cardiac output, PAC pulmonary artery catheter, TTE transthoracic echocardiography, TOE trans-oesophageal echocardiography, Vigileo: Vigileo ™ (Edwards Lifesciences, California, USA).

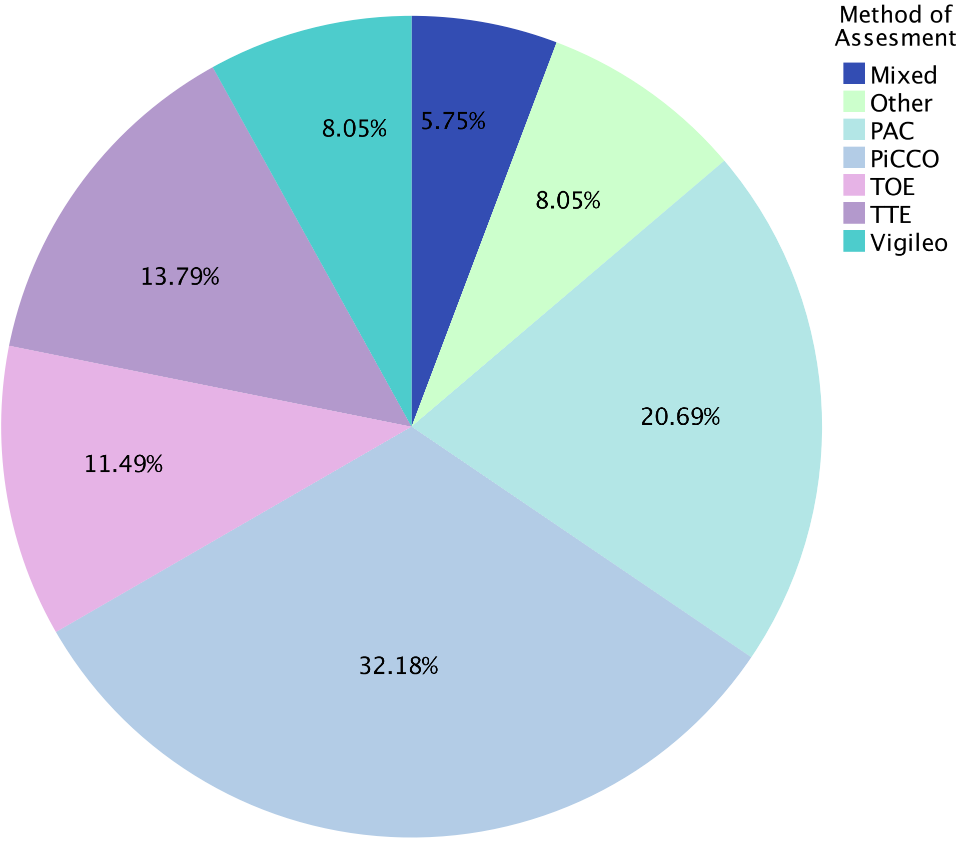

eFigure 3. Comparison of the proportion of responders (%) by volume of fluid used for the fluid challenge. Columns level represent means and error bars represent 95% confidence intervals.

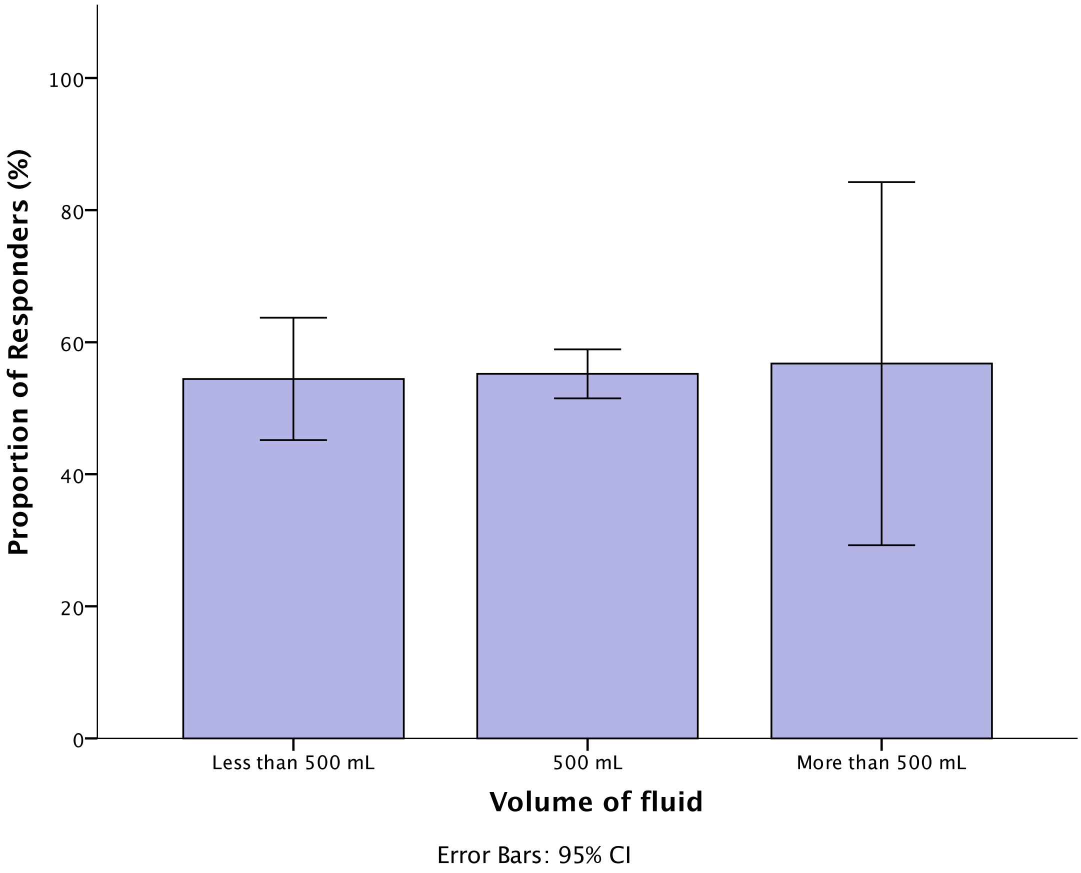

eFigure 4. Comparison of the proportion of responders (%) by type of fluid used for the fluid challenge. Columns level represent means and error bars represent 95% confidence intervals.

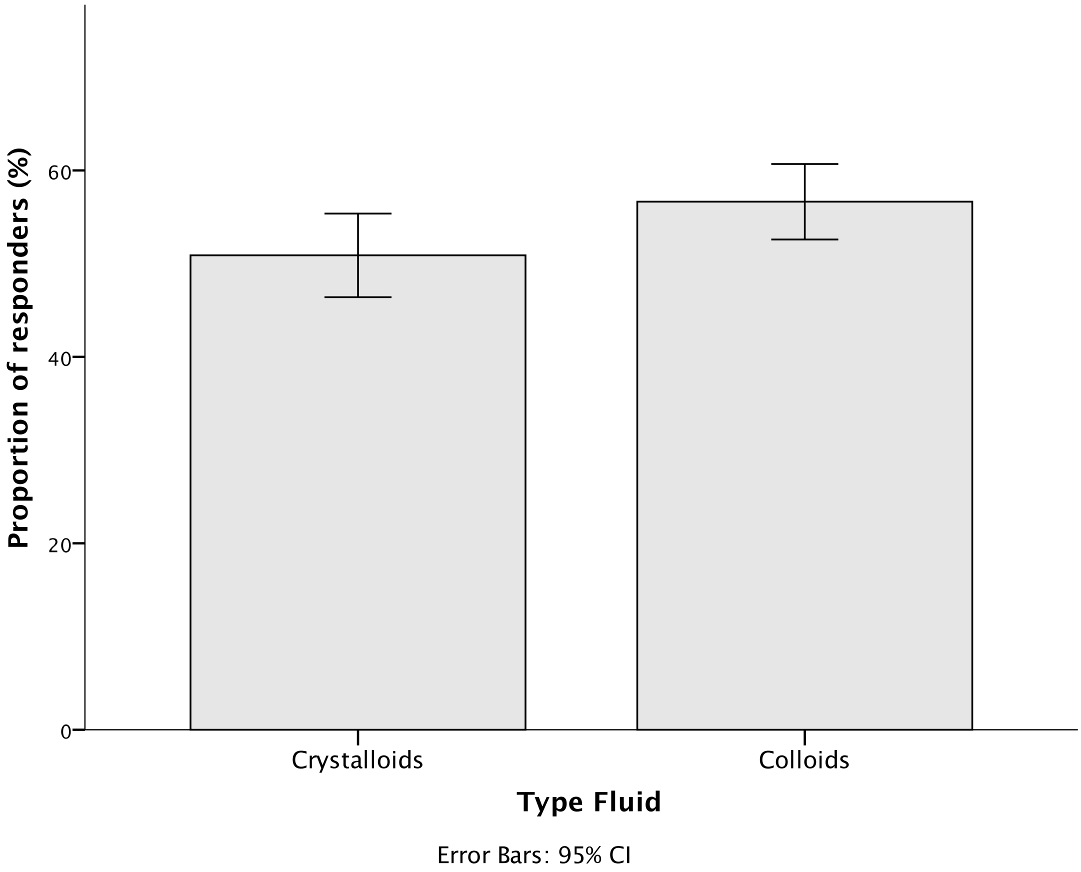

Supplement: Additional file 1: — Study protocol, PRISMA checklist and additional figures. (PDF 674 kb) [file 13054_2017_1796_MOESM1_ESM.pdf]
